# Multisystemic Tuberculosis Masquerading as Aggressive Cardiac Tumor Causing Budd–Chiari Syndrome Disseminated to the Brain Resulting in Death of a Six-Year-Old Boy

**DOI:** 10.3390/pathogens14080772

**Published:** 2025-08-05

**Authors:** Eman S. Al-Akhali, Sultan Abdulwadoud Alshoabi, Halah Fuad Muslem, Fahad H. Alhazmi, Amirah F. Alsaedi, Kamal D. Alsultan, Amel F. Alzain, Awatif M. Omer, Maisa Elzaki, Abdullgabbar M. Hamid

**Affiliations:** 1 Department of Radiology, Al Anwar Medical Hospital, Hail 55424, Saudi Arabia; 2 Department of Radiology, Advanced AlRazi Diagnostic Center, Sana’a 999101, Yemen; 3 Department of Diagnostic Radiology, College of Applied Medical Sciences, Taibah University, Al-Madinah Al-Munawwarah 42353, Saudi Arabia; 4 Department of Internal Medicine, Dr. Suliman Al Habib Hospital Altakhasosi, Riyadh 12344, Saudi Arabia; 5 Radiology Department, Rush University Medical Group, Chicago, IL 60612, USA

**Keywords:** tuberculosis, right atrial mass, tree-in-bud sign, budd–chiari syndrome, nutmeg appearance of the liver, brain tuberculomas

## Abstract

Tuberculosis (TB) is an ancient and re-emerging granulomatous infectious disease that continues to challenge public health. Early diagnosis and prompt effective treatment are crucial for preventing disease progression and reducing both morbidity and mortality. These steps play a vital role in infection control and in lowering death rates at both individual and population levels. Although diagnostic methods have improved sufficiently in recent decades, TB can still present with ambiguous laboratory and imaging features. This ambiguity can lead to diagnostic pitfalls and potentially disastrous outcomes due to delayed diagnosis. In this article, we present a case of TB that was difficult to diagnose. The disease had invaded the mediastinum, right atrium, right coronary artery, and inferior vena cava (IVC), resulting in Budd–Chiari syndrome. This rare presentation created clinical, laboratory, and radiological confusion, resulting in a diagnostic dilemma that ultimately led to open cardiac surgery. The patient initially presented with progressive shortness of breath on exertion and fatigue, which suggested possible heart disease. This suspicion was reinforced by computed tomography (CT) imaging, which showed infiltrative mass lesions predominantly in the right side of the heart, invading the right coronary artery and IVC, with imaging features mimicking angiosarcoma. Although laboratory findings revealed an exudative effusion with lymphocyte predominance and elevated adenosine deaminase (ADA), the Gram stain was negative for bacteria, and an acid-fast bacilli (AFB) smear was also negative. These findings contributed to diagnostic uncertainty and delayed the confirmation of TB. Open surgery with excisional biopsy and histopathological analysis ultimately confirmed TB. We conclude that TB should not be ruled out solely based on negative Mycobacterium bacteria in pericardial effusion or AFB smear. TB can mimic aggressive tumors such as angiosarcoma or lymphoma with invasion of the surrounding tissues and blood vessels. Awareness of the clinical presentation, imaging findings, and potential diagnostic pitfalls of TB is essential, especially in endemic regions.

## 1. Introduction

Tuberculosis (TB) is a chronic infectious disease mainly caused by *Mycobacterium tuberculosis* [1]. It is the second leading cause of death from a contagious disease worldwide [1,2]. According to the World Health Organization Global TB Report 2022, there are more than 10,000,000 new TB cases and approximately 1,300,000 TB-related deaths annually worldwide. Around 30% of TB cases are either underdiagnosed or underreported to heath authorities, contributing to an underestimation of TB-related mortality [3]. While the lungs are the primary site of TB infection, the disease can also affect extrapulmonary organs such as lymph nodes, pleura, intestines, brain, bones, and joints. This form is referred as extrapulmonary TB [2,4].

Despite recent advances in TB diagnosis, laboratory tests remain limited or unavailable in resource-constrained settings where TB is most prevalent [5]. Extrapulmonary TB can present with nonspecific symptoms, such as fever of unknown origin, making diagnosis even more challenging. Medical imaging modalities including conventional radiography, computed tomography (CT), ultrasonography, magnetic resonance imaging (MRI), and positron emission tomography modalities play a crucial role in detecting tissue abnormalities and raising early suspicion of TB. With experienced radiologists, these imaging modalities can aid in diagnosing various forms of TB including pulmonary, spinal, cerebral, and abdominal TB [6].

In addition to being familiar with the clinical manifestations of TB, radiologists, chest physicians, and infectious disease specialists should also be aware of the typical imaging findings of both pulmonary and extrapulmonary TB. This article presents a very rare and complex case that was difficult to diagnose based on clinical symptoms, laboratory tests, and even a sequence of medical imaging studies including chest radiography, CT, and MRI. The final diagnosis was made only after open surgery and histopathological examination of an excisional biopsy. This case underscores the fact that TB can mimic malignant cardiac tumors, leading to significant diagnostic dilemmas and potentially unnecessary or high-risk surgical interventions.

## 2. Case Presentation

A 6-year-old male patient presented with progressive shortness of breath on exertion and fatigue for 10 days. On clinical examination, the patient was conscious, oriented, and febrile. His fetal signs were as follows: temperature 38.2 °C, heart rate 110–130 beats per minute, respiratory rate 24–30 breaths per minute, blood pressure 90/60 mmHg, and oxygen saturation 92–95% on room air. Initial chest imaging revealed a massive pericardial effusion with mild left-side pleural effusion.

**Figure 1:**
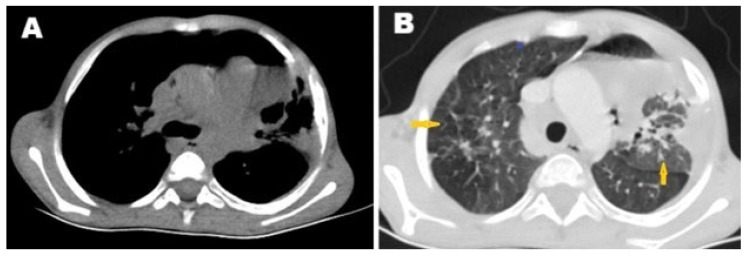
Selected axial images of computed tomography of (**A**) chest window, showing left lung lesion and (**B**) lung window, showing left lung consolidation with numerous centrilobular nodules with opacified distal bronchioles forming the tree-in-bud sign (arrows) with minimal left pleural effusion.

**Figure 2:**
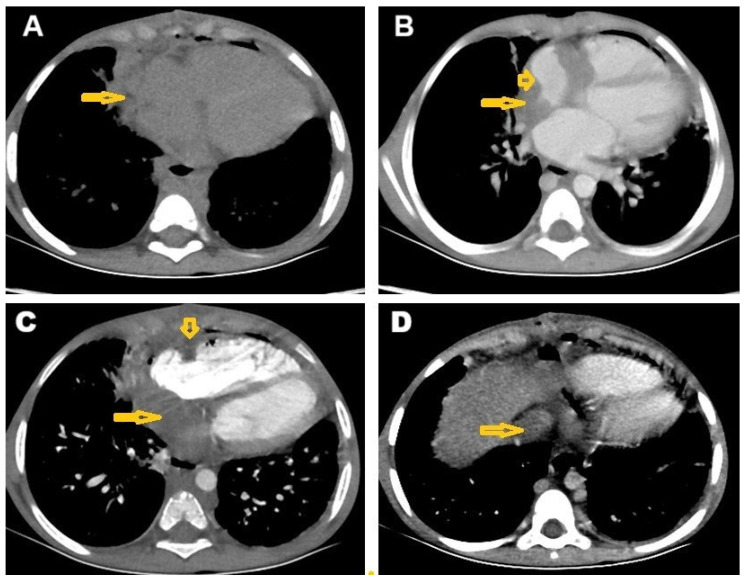
Selected cardiac computed tomography images. (**A**) Plain axial CT mediastinal window showing ill-defined right mediastinal infiltrative lesion around the right heart wall, and (**B**) post-contrast axial CT showing right mediastinal enhanced infiltrative lesion (arrows) around the right heart border that infiltrate the right atrium (short arrow). (**C**) Post-contrast axial CT mediastinal window showing ill-defined right mediastinal infiltrative lesion (arrow) around the right heart wall involving the right coronary artery (short arrow), and (**D**) post-contrast axial CT showing filling defect in the inferior vena cava (IVC) as a consequence of infiltration (arrows).

Chest CT revealed numerous centrilobular nodules with opacified distal bronchioles forming a tree-in-bud sign, as well as minimal left pleural effusion (Figure 1). Additionally, both CT and MRI showed an infiltrative lesion in the right mediastinum that involved the right side of the heart (Figure 2A,B), invading the right coronary artery (Figure 2C), and associated with thrombosis of the inferior vena cava (IVC) (Figure 2D).

**Figure 3:**
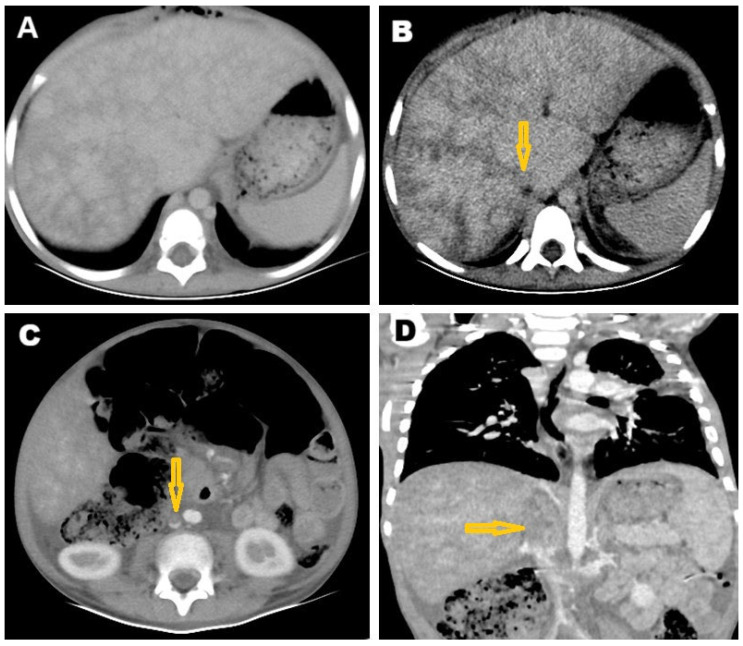
Selected images from triphasic liver computed tomography study. (**A**) Post-contrast venous phase axial CT of the liver showing mottled appearance of the liver due to congestion, likely Budd–Chiari syndrome; (**B**) post-contrast delayed-phase axial CT of the liver showing heterogeneous liver density with infiltration of the IVC (arrow); (**C**) post-contrast axial CT showing filling defect of the inferior vena cava (IVC) as a consequence of infiltration (arrow) down to the lower abdomen; and (**D**) post-contrast coronal reconstruction CT confirming IVC infiltration inside the liver (arrow).

Abdominal CT showed heterogeneous liver density with infiltration of the IVC (Figure 3A). The post-contrast CT revealed a mottled appearance of the liver, indicative of congestion and suggestive of Budd–Chiari syndrome (Figure 3B). Additionally, a filling defect within the IVC extending to the lower abdomen was also observed (Figure 3C). IVC obstruction was confirmed on post-contrast coronal reconstruction CT (Figure 3D). The CT findings suggest the presence of an infiltrative malignant tumor, likely lymphoma, or, alternatively, atypical cardiac myxoma or granulomatous infection—possibly TB—as differential diagnoses associated with Budd–Chiari syndrome. The thrombotic occlusion of the IVC is a typical finding used to diagnose Budd–Chiari syndrome.

Following CT, the pericardial fluid was aspirated and the breathing was improved. Tuberculin skin test was negative. Both AFB smear and bacterial culture of pericardial effusion yielded negative results. Unfortunately, specific culture for Mycobacterium tuberculosis, either liquid or solid, was intermittently available in the Tuberculosis Center in Sana’a and testing could not be carried out. A true cut biopsy was conducted and the histopathology results showed normal tissue. The patient was treated with low-molecular-weight Heparin, Furosemide, and antibiotics until improved.

**Figure 4:**
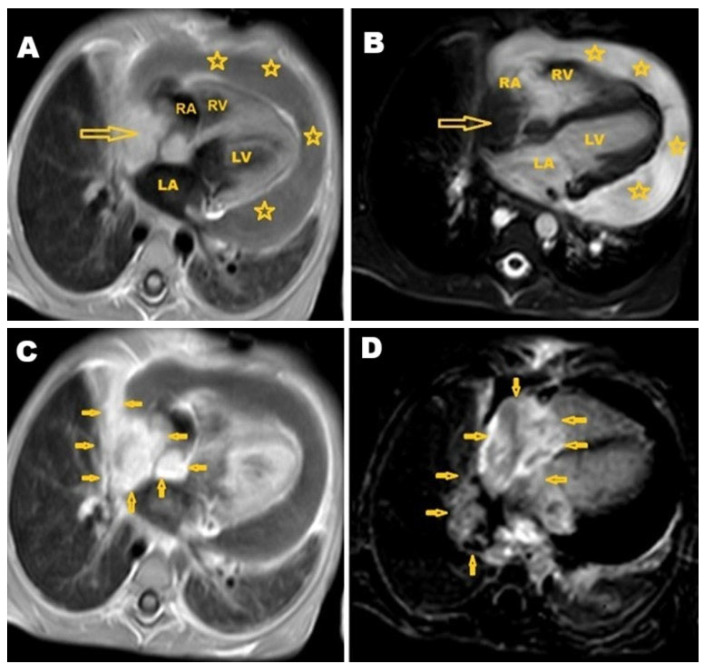
Selected cardiac MRI images. (**A**) Axial T1 4-chamber view shows isointense infiltrative lesion in the right heart wall (arrow) invading the right atrium (RA) with a large amount of low-signal-intensity pericardial effusion (stars); (**B**) T2 4-chamber view shows a hypointense mass lesion in the right wall of the heart (arrow) invading the RA, with a large amount of high-signal-intensity pericardial effusion (stars); (**C**) T1 post-contrast 4-chamber view shows large ill-defined mass lesion infiltrating the right side of the mediastinum, invading the right pericardium, right atrial wall, and interatrial septum, and associated with large pericardial effusion; and (**D**) late gadolinium enhancement (LGE) 4-chamber view shows enhancement of the lesion. MRI: magnetic resonance imaging, RA: right atrium, LA: left atrium, RV: right ventricle, LV: left ventricle.

**Figure 5:**
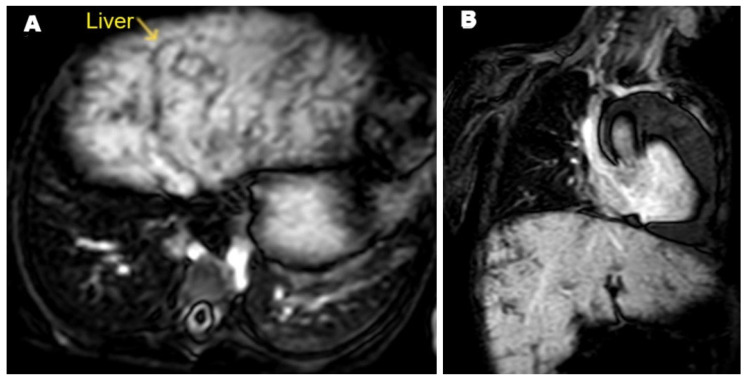
Selected late gadolinium enhancement (LGE) MRI images. (**A**) Axial and (**B**) coronal sections showing contrast reflux into the hepatic veins, causing nutmeg appearance of the liver and confirming the diagnosis of Budd–Chiari syndrome.

After five weeks, shortness of breath and significant weight loss was noted with no cough, no fever, no headache, and no vomiting. On clinical examination, the patient was alert and conscious, with no signs of central nervous system problems. Slight bilateral lower limb edema was noted. The diagnosis remained unclear, prompting a referral for cardiac MRI. The cardiac MRI was carried out at the Advanced AlRazi Diagnostic Center, Sana’a, Republic of Yemen, which revealed an infiltrative mass lesion in the right heart wall that was invading the right atrium (RA). This mass demonstrated intermediate signal intensity on T1-weighted images and low signal intensity on T2-weighted images. In addition, a significant amount of pericardial effusion was observed around the heart, showing low signal intensity on T1-weighted images (Figure 4A) and high signal intensity on T2-weighted images (Figure 4B). The mass exhibited enhancement on T1 post-contrast images (Figure 4C), and also showed late gadolinium enhancement (LGE) (Figure 4D). The MRI findings indicated a malignant infiltrative mass lesion invading the right coronary artery, consistent with angiosarcoma, as suggested by the CT. Furthermore, the axial LGE MRI showed a nutmeg appearance of the liver, confirming the diagnosis of Budd–Chiari syndrome (Figure 5).

Therapeutic and diagnostic pericardial fluid aspiration was performed. The analysis of the pericardial fluid indicated elevated protein levels, low to normal glucose, elevated lymphocyte, and high adenosine deaminase (ADA). The Gram stain result was negative for bacteria, and the acid-fast bacilli (AFB) smear was negative. Additionally, cytology did not reveal any malignant cells. Overall, both AFB smear and bacterial culture yielded negative results, which led to confusion. For a definitive diagnosis, it was recommended that the clinical findings be correlated with imaging and histopathological examination.

**Figure 6:**
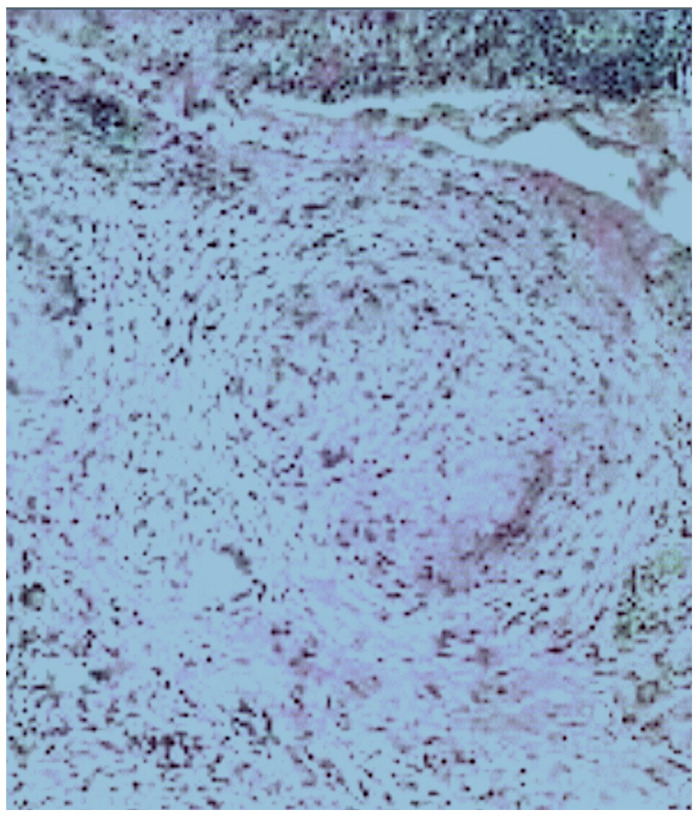
Selected microscopic image of white tissue sample from right atrial mass showing chronic inflammatory tissue infiltrating the right atrial wall, with aggregations of small lymphocytes and the formation of epithelioid granulomas with a central collection of neutrophils and fibrosis. The histopathological finding of a necrotizing granulomatous inflammatory reaction strongly suggested tuberculosis.

After 8 weeks, due to the unclear nature of the lesion and its predominant malignant behavior, as described above, an open cardiac surgery was performed through a median sternotomy. This revealed a right mediastinal mass invading the right atrium (RA), right pulmonary veins, and IVC. In addition, pericardial and lung adhesions were found. The adhesions were carefully dissected, and an incisional biopsy was taken from the RA. Histopathological analysis confirmed necrotizing granulomatous inflammation, likely due to TB (Figure 6) affecting both the epicardium and right atrium. This condition resulted in thrombosis of the right coronary artery and IVC. A retrospective review of the previous pulmonary imaging findings indicated the endobronchial spread of TB.

The patient was started on a full anti-TB treatment protocol as follows: Isoniazid (INH) 10 mg/kg/day (maximum 300 mg), Rifampicin (RIF) 15 mg/kg/day (maximum 600 mg), Pyrazinamide (PZA) 35 mg/kg/day (maximum 2000 mg), and Ethambutol (EMB) 20 mg/kg/day (maximum 1200 mg). Additionally, the patient received Prednisolone at a dosage of 1–2 mg/kg/day, typically prescribed for 4 weeks. Following treatment initiation, there was slight improvement in the pericardial effusion.

**Figure 7:**
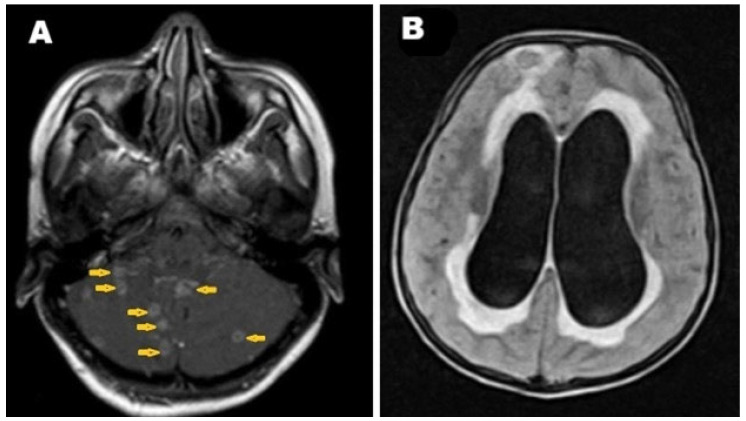
Selected brain MRI images. (**A**) Post-contrast T1-weighted image shows multiple intracranial ring-enhanced lesions (arrows) involving the cerebellum, strongly suggesting tuberculous foci, with perifocal edema causing mass effect upon the fourth ventricle. (**B**) FLAIR-weighted image shows obstructed hydrocephalus with active permeation appearing as a high signal intensity around the ventricles.

Two weeks after surgery, the patient developed a severe headache, prompting a brain MRI. The MRI revealed multiple intracranial ring-enhanced lesions involving the cerebellum, likely representing tuberculous foci (Figure 7A), and confirming brain involvement. Furthermore, obstructive hydrocephalus with active cerebrospinal fluid permeation was identified (Figure 7B). A diagnosis of brain TB complicated by obstructive hydrocephalus was made, and the patient was prepared for ventriculoperitoneal shunt (VP-shunt).

On the day 16 after brain MRI, despite anti-TB treatment, the patient’s condition deteriorated before undergoing VP-shunt placement, and unfortunately, the patient passed away. The final diagnosis was disseminated TB, pulmonary and extrapulmonary TB, involving the mediastinum, heart, IVC, and brain.

## 3. Discussion

TB is an ancient and re-emerging chronic granulomatous infectious disease that continues to challenge public health. Early diagnosis and the prompt initiation of effective anti-TB treatments are essential to prevent progression to severe or disseminated disease and to reduce both morbidity and mortality. Timely diagnosis also plays a crucial role in infection control and in decreasing mortality at both individual and public health levels. In this article, we reported a very rare case of TB that masqueraded as an aggressive malignant cardiac tumor, leading to open-heart surgery and ultimately resulting in the patient’s death. This case is consistent with a previously reported case of multisystemic TB presenting as coma due to tuberculous meningitis, vasculitis, and pericarditis in a young adult male patient [7].

Extrapulmonary TB often presents with nonspecific clinical manifestations, making diagnosis challenging. In the current case, the patient experienced progressive shortness of breath during exertion and fatigue, which initially suggested heart disease. CT imaging supported this suspicion by revealing infiltrative mass lesions predominantly affecting the right heart and invading the right coronary artery and IVC. Previous studies have shown that primary cardiac tumors are exceedingly rare, with 70–80% being benign and 20–30% being malignant, such as angiosarcoma, lymphoma, fibrosarcoma, and myosarcoma. Angiosarcoma, in particular, is associated with poor survival rates due to early metastasis, and radical surgical resection remains the most effective approach for improving outcomes [8]. In the current case, the lesion was located in the RA, which is the most common site of primary cardiac angiosarcoma, as noted by Kiwaki et al. and Yu et al. [9,10]. The lesion was infiltrative, invading the right mediastinum, heart wall, and blood vessels, with non-specific symptoms, findings that were also consistent with cardiac lymphoma [11,12].

The first step in evaluating pericardial effusion is determining whether it is a transudate or an exudate. Transudates usually indicate systemic disease, while an exudate suggests pericardial infection, such as TB or malignancy. The gold standard for diagnosing pleural TB is the identification of Mycobacterium tuberculosis in pericardial fluid or tissue [13]. In the current case, the laboratory findings were consistent with an exudative effusion, with a predominance of lymphocytes and elevated ADA suggestive of tuberculous pericarditis. However, Gram staining was negative for bacteria, and the AFB smear was also negative, which complicated the diagnosis of TB. This situation is not uncommon in paucibacillary forms of tuberculosis. Although elevated ADA levels of more than 40 IU/L show good diagnostic accuracy for pericardial TB, AFB can be identified in 0–42% in pericardial fluid samples and approximately 10–70% of pericardial tissue biopsies [14].

The diagnosis of malignancy from pericardial effusion is based on the detection of malignant cells in either pericardial effusion or pericardial tissue biopsy. The imaging features of CT and cardiac MRI overlap with inflammatory and infectious pericarditis [15]. In the present patient, cytological analysis revealed no malignant cells. The correlation of this finding with medical imaging suggested the diagnosis of a benign tumor, specifically an atypical cardiac myxoma. A recent study reported that CT attenuation has incremental diagnostic value in differentiating malignant from benign pericardial effusion [16]. In our patient, the CT findings suggested a diagnosis of TB; however, the MRI results caused confusion. The confusion of medical imaging can be explained by the aggressive and infiltrative nature of the lesion in the current patient, which had invaded the mediastinum, right heart wall, right atrium, right coronary artery, and IVC.

Ultimately, TB can masquerade as aggressive malignant tumors, causing diagnostic misunderstandings and potentially disastrous outcomes. Numerous reports in the literature have documented TB mimicking malignancy across various organ systems. TB has been reported to present as a lung mass simulating metastatic lung cancer [17], and as a solitary cerebellar tuberculoma mimicking central nervous system tumors based on clinical manifestations and medical imaging features [18]. Cases have also been documented where solitary cerebral tuberculomas mimicked glioblastoma multiforme, prompting neurosurgical intervention in an adult patient [19] and another case in a child [20]. TB has also been reported as spinal cord tuberculomas imitating intramedullary metastasis [21], and as a nasal mass in the right middle meatus that bled on contact in a 24-year-old man, creating diagnostic challenges [22]. Another report described TB presenting as paraspinal muscles mass in an 18-year-old male [23].

Hepatic TB, though rare, may mimic liver malignancies [24]. Pancreatic TB may mimic pseudocyst or pancreatic carcinoma, sometimes resulting in unnecessary and potentially harmful surgery [25]. TB was also reported to mimic a spindle cell tumor of the proximal esophagus in a 24-year-old man [26], and as an abdominal mass with clinical features suggestive of colorectal cancer [27]. Peritoneal TB is among the most diagnostically challenging forms, often mimicking peritoneal carcinomatosis [28]. It was also described as presenting with peritoneal thickening and ovarian enlargement with masses mimicking ovarian malignancy in a 16-year-old female [29], and as a compressive pelvic mass with secondary hydronephrosis in a 20-year-old female [30]. TB was reported to masquerade as endometriosis in a 23-year-old female presenting with acute abdomen [31], and even as an isolated prostatic lesion resembling prostate cancer [32]. These cases illustrate that TB can present with a wide range of clinical manifestations, mimic numerous pathological entities, and affect virtually any organ in the body.

**Limitations:** This case report is limited by the lack of detailed patient history prior to referral to the radiology center, as well as the absence of the initial chest radiograph and abdominal ultrasonography images. Unfortunately, specific culture for Mycobacterium tuberculosis was difficult to obtain in the developing, unstable country of Yemen. In addition, Xpert MTB/RIF Ultra or other modern nucleic acid amplification tests (NAATs) were not available. The patient was left at home during periods of improvement with no full details about these periods being obtained.

## 4. Conclusions

TB can present with a wide range of clinical manifestations, affect any organ system, and exhibit diverse imaging and laboratory features, making diagnosis highly challenging. TB may mimic aggressive malignancies such as angiosarcoma or lymphoma, with the invasion of surrounding tissue and vascularity structures. Importantly, the possibility of TB should not be ruled out solely based on negative tests for Mycobacterium tuberculosis bacteria in pericardial effusion or a negative AFB smear. Awareness of TB’s variable clinical presentations, imaging characteristics, and diagnostic pitfalls is essential—particularly in regions where TB is endemic—to ensure timely and accurate diagnosis and avoid unnecessary or harmful interventions.

## Data Availability

The original contributions presented in this study are included in the article. Further inquiries can be directed to the corresponding authors.

## Abbreviations

The following abbreviations are used in this manuscript.

ADA	adenosine deaminase
AFB	acid-fast bacilli
CT	computed tomography
IVC	inferior vena cava
MRI	magnetic resonance imaging
RA	right atrium
TB	tuberculosis
